# Response of fermentation quality and microbial community of oat silage to homofermentative lactic acid bacteria inoculation

**DOI:** 10.3389/fmicb.2022.1091394

**Published:** 2023-01-20

**Authors:** Muhammad Tahir, Jiayi Li, Yafen Xin, Tianwei Wang, Chen Chen, Yihao Zhong, Lei Zhang, Haiping Liu, Yaling He, Xingjin Wen, Yanhong Yan

**Affiliations:** ^1^College of Grassland Science and Technology, Sichuan Agricultural University, Chengdu, China; ^2^State Key Laboratory of Microbial Resources, Institute of Microbiology, Chinese Academy of Sciences, Beijing, China; ^3^School of Life Sciences, University of Chinese Academy of Sciences, Beijing, China

**Keywords:** oat, lactic acid bacteria, chemical characteristics, silage quality, microbial community

## Abstract

Oat (*Avena sativa* L.) is one of the important forage crops in the world. However, oat grown in Southwest China has higher moisture content and their preservation face significant challenges. In addition, existing commercial lactic acid bacteria (LAB) have poor fermentation effects in hot and humid regions. Consequently, the current study investigated the response of oat fermentation quality and microbial community to self-selected LAB inoculation. The treatments were: CK, sterilized water; LP694, *Lactobacillus plantarum* 694; LR753, *Lactobacillus rhamnosus* 753; and LPLR, LP694 combined with LR753, followed by 1, 3, 7, 14, and 60 days (d) of fermentation. The results showed that LAB inoculation significantly raised the lactic acid content, and decreased the level of pH value, acetic acid, and ammonia-N in oat silage. The LR753 group had a significantly higher (*p* < 0.05) lactic acid content (60.95 g kg^–1^ DM), and lower pH value (3.95) and ammonia-N content (10.1 g kg^–1^ DM) followed by the LPLR group. The LR753 showed lower NDF (54.60% DM) and ADF (39.73% DM) contents than other groups. The *Lactobacillus* was a prevalent genus in LAB-treated groups, and its relative abundance reached maximum in LP694 (69%) on day 3, while in the LR753 group (72%) on 60 days. The *Lactobacillus rhamnosus, Lactobacillus plantarum*, and *Lactobacillus fermentum* became the dominant species in LAB-treated groups with fermentation time. The *Lactobacillus* genus was positively correlated with WSC (*R* = 0.6, *p* < 0.05), while negatively correlated with pH (*R* = −0.5, *p* < 0.05), and BA (*R* = −0.5, *p* < 0.01). Overall, the LR753 group had better fermentation quality and preservation of nutritional components providing theoretical support and guidance for future oat silage production in Southwest China.

## Graphical Abstract

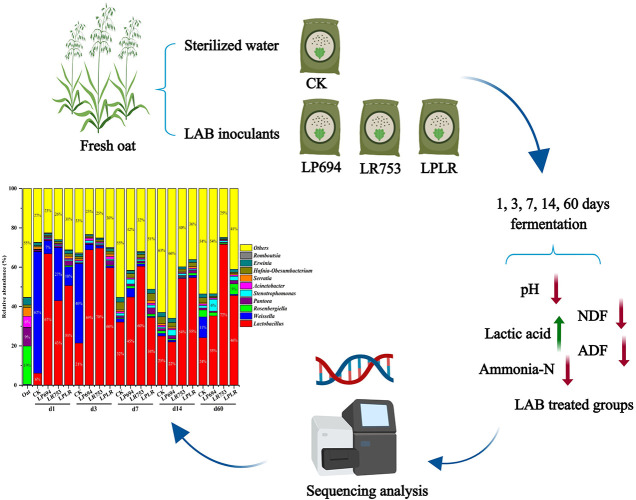

## 1. Introduction

Oat (*Avena sativa* L.) is one of the most important forage and food crops worldwide which is characterized by greater tolerance to the saline-alkaline environment, drought, cold, and nutrient-deficiency stresses (Reynolds and Suttie, 2004; Andrzejewska et al., 2019). Recently, oat forage has emerged as one of the major crops in China due to its greater biomass production and feeding value, is widely cultivated in Northern, Northwestern, and Southwestern China (Diao, 2017). Utilization of oat forage as silage is an effective method to guarantee year-round access to good-quality silage for ruminants (Cheng et al., 2022). However, the oat that grows in Southwest China has a higher moisture content, making its preservation significantly challenging due to the hot and humid conditions.

Ensiling is a traditional fresh forage preservation practice to overcome the variance between livestock production and the seasonal unavailability of forages (Yang et al., 2022). Ensiling plays a vital role to help livestock survive winters and dry seasons in many countries around the world by conservation of fresh forage (Xia et al., 2022). During the ensiling process, lactic acid bacteria (LAB) cause rapid acidification under anaerobic conditions by converting water-soluble carbohydrates (WSC) into organic acids, mainly lactic acid (LA) by which the growth of spoilage microorganisms is restrained (Zi et al., 2021). However, oat ensiling involves several factors, which may have individual or interactive effects (such as the moisture content of raw materials, type of microorganisms, and additives used in fermentation) on the silage quality (Zhang et al., 2018). High-moisture silages often bear a high risk of effluent production leading to dry matter (DM) loss (Gebrehanna et al., 2014). Xu et al. (2022) reported that the appropriate moisture content might help produce high-quality oat silage and provide a simple and feasible method to enhance the effects of bacteria and enzymes. However, in this study, the oat was wilted first, adjusted to proper moisture content, and then ensiled with different additives. Hence, it is important to ensile oat forage directly after harvesting rather than wilting first to understand its fermentation mechanism which could save time, labor, and money.

The LAB additives are often used to improve the fermentation quality of silages (Wang T. et al., 2020; Xia et al., 2022). LAB inoculants isolated from different crops or regions have different fermentation patterns, and their impacts are usually substrate and environment-dependent (Zhang et al., 2015). Therefore, the selection and application of LAB to produce different types of silages are more critical. Our previous studies reported that *Lactobacillus plantarum* 694 (LP694) isolated from high-moisture corn silage in Southern China could change the microbial diversity and influence the fermentation quality of high-moisture Italian ryegrass (Yan et al., 2019), and *Lactobacillus rhamnosus* 753 (LR753) isolated from corn silage in the subtropical region could prolong the aerobic stability of corn silage in the tropical and subtropical areas (Guan et al., 2020). Since oat is mainly grown in winter and spring seasons, how the LAB strains screened in hot and humid regions influence the fermentation pattern of oat forage remains unclear. Moreover, the microorganisms in silage play a critical role in the fermentation process. Monitoring the changes in the bacterial community during fermentation gives an insight into understanding and improving the ensiling process (He et al., 2020). In essence, ensiling fermentation is a competitive process between LAB and undesirable microorganisms, and more attention is valuable for the succession of the bacterial community that dictates silage quality.

Consequently, the current study aimed to investigate the response of fermentation quality and microbial community succession of oat to self-selected native LAB inoculations. The results of this study may provide theoretical support and guidance for future oat silage production in Southwest China with the help of suitable LAB inoculants, supporting the current shifts toward sustainable and high-efficiency agricultural production systems.

## 2. Materials and methods

### 2.1. Materials and silage preparation

The oat forage (cultivar Menglong) was harvested at the late heading stage from Modern Agriculture Research and Development Base of Sichuan Agricultural University, Chongzhou, China (103°07′ E, 30°30′ N) on 10 May 2021. The two self-selected LAB strains–LR753 and LP694–which were used as additives for silage preparation were isolated in our laboratory. LR753 and LP694 have been accessioned at the China General Microbiological Culture Collection Center under CGMCC Nos. 18233 and 15073, respectively. The harvested forage was chopped into 2.0 cm by a crop chopper (ZS-2, Zhongsheng agricultural machinery company, Tangshan, China). The LAB treatments included LP694, LR753, and LP694 in combination with LR753 (LPLR, 1:1). Each LAB strain was incubated by using De Man, Rogosa, Sharpe agar (MRS) broth (CM 188, Land Bridge, Beijing, China) and dissolved in sterile distilled water to an equivalent of 10^6^ colony-forming units (cfu)/g of fresh matter (FM). An equal amount of sterilized water was prepared to set a control group (CK). The prepared LAB inoculants and sterilized water was sprayed on the fresh chopped oat. About 300 g of prepared forages were packed into vacuum-sealed polyethylene plastic bags (dimensions 225 mm × 350 mm, Aodeju, Sichuan, China) and vacuum-sealed with a vacuum machine (DZ-AS, 2500KW, ANSEN, Fujian, China). A total of 60 bags (4 treatments ×5 ensiling days ×3 replicates) were conserved at room temperature. The silage samples were obtained at 1, 3, 7, 14, and 60 days (d) of ensiling to evaluate the chemical composition, fermentation quality, and microbial community.

### 2.2. Chemical and fermentation profile analysis

The chemical and fermentation characteristics analysis was performed according to the previously described method by Zeng et al. (2020). Briefly, the pre- and post-ensiling samples (200 g) were oven dried at 65°C till constant weight for DM content determination and then ground to pass a 1 mm screen for chemical component analysis. The WSC concentration was determined by the thracenone-sulphuric acid method, while that of crude protein (CP) concentration was measured by the Kjeldahl method (Association of Official Analytical Chemists [AOAC], 1990). The neutral detergent fiber (NDF) and acid detergent fiber (ADF) contents were determined by the methods described by Van Soest et al. (1991).

About 20 g of silage sample and 180 ml sterilized water were blended for 24 h at 4°C and then passed through four layers of gauze to analyze the pH values, ammonia-N, and the organic acids, including LA, acetic acid (AA), propionic acid (PA), and butyric acid (BA). The pH value of the resulting extract was instantly analyzed using a portable pH meter (PHSJ-5; LEICI, Shanghai, China). Part of the water extract was centrifuged for 10 min at 12,000 × *g* at 4°C for organic acids measurement and passed through 0.22 μm membrane *via* high-performance liquid chromatography (HPLC, KC-811, Shimadzu Co., Ltd., Kyoto, Japan). The HPLC was equipped with a UV detector setting a detection wavelength of 210 nm. About 3 mmol/L perchloric acid (0.5 mL/min) was used as the mobile phase at 55°C. The ammonia-N (AN) content was determined using the ninhydrin colorimetric and phenol-hypochlorite method (Broderick and Kang, 1980).

### 2.3. Cultured-based microbial analysis

The plate count method was used for microbial analysis (Zeng et al., 2020). Undried pre- and post-ensiling samples (20 g) were homogenized with 180 ml of the sterilized saline (0.85% w/v NaCl) by blending thoroughly. The homogenized solution was diluted continuously from 10^0^ to 10^–5^ after filtration with a single-layer sterilized gauze. In a sterilized environment, the filtrate was inoculated on MRS (Land Bridge, Beijing, China), Violet Red Bole Agar (VRBA, Land Bridge, Beijing, China), and Potato Dextrose Agar (PDA, Land Bridge, Beijing, China) to count the number of LAB, coliform bacteria, and yeasts/molds, respectively (Zeng et al., 2020). The LAB plates were incubated under anaerobic envrionment at 37°C for 48 h, the VRBA plates were incubated under aerobic conditions at 37°C for 24 h, and the PDA plates were aerobically incubated at 25°C for 4 d. All microbial populations were measured as cfu/g of FM and were then log-transformed.

### 2.4. Bacterial community analysis

The method of DNA extraction was referred from Guan et al. (2018)—the samples (20 g) were shaken in 180 ml of sterile saline (0.85% NaCl) for 30 min at 4°C, filtered through two-layer medical gauze, and then centrifuged at 10,000 × *g* for 15 min at 4°C. The supernatant was discarded, and the pellet was used for DNA extraction. The TIANamp bacterial DNA extraction kit (DP302-02, Tiangen, Beijing, China) was used for total DNA extraction. The quality and purity of the extracted DNA were analyzed by 1% agarose gel electrophoresis and spectrophotometry (260/280 nm). The DNA concentration for all samples was adjusted to 1 ng μl^–1^. The qualified DNA samples were stored at −20°C until subsequent analysis.

The V4 region of bacterial 16S rDNA gene was amplified using specific primers, 515F (5′-GTTTCGGTGCCA GCMGCCGCGGTAA-3′) and 806R (5′-GCCAATGGACTACHV GGGTWTCTAAT-3′). The PCR products were analyzed by electrophoresis using 2% agarose gel, and the qualified PCR products were further purified by magnetic beads and quantified by enzyme labeling. The purified samples were mixed thoroughly at an equal amount (determined based on the concentration of PCR products) before loading onto 2% agarose gel. The PCR product was detected by glycogen electrophoresis, and the target band was recovered using a gel recovery kit.

The TruSeq^®^ DNA PCR-Free Sample Preparation Kit was used for library construction. The constructed library was quantified by Qubit and q-PCR. After the library was quantified, it was then subjected to on-machine sequencing using a NovaSeq6000 sequencer. The data were analyzed using the Novogene Magic Platform. The low-quality reads were removed using Cutadapt (V1.9.1),^1^ following a previous study (Martin, 2011). Sample data were split from obtained reads according to the barcode, then the barcode and primer sequences were cut off according to the reads from the barcode to obtain fresh reads, and the chimera sequence was removed to get the valid data. All clean reads of all samples were clustered by using Uparse software (uparse v7.0.1001) following a previous study (Rognes et al., 2016).^2^ The operational taxonomic units (OTUs) were defined with a similarity cutoff of 97%. The OTUs sequence was annotated, and the species annotation was analyzed with the Mothur method and the SSUrRNA database (Edgar, 2013) of silva132 (Haas et al., 2011)^3^ to obtain the taxonomic information. The Shannon, Simpson, Chao 1, ACE, and Goods coverage indexes were calculated by QIIME software (version 1.7.0).

### 2.5. Statistical analysis

All the reported results are the mean of three replicates, and the data were analyzed using the SPSS software (Version 28.0: IBM Corp., Armonk, NY). The chemical composition, fermentation quality, and alpha diversity were analyzed using a two-way analysis of variance with Duncan’s multiple-range test. The relationships between the bacterial taxonomic profile and silage quality variables were determined by calculating the spearman correlation coefficients and were plotted by using the “pheatmap” libraries in R. The significance was employed at a 0.05 probability level to compare the means.

## 3. Results

### 3.1. Characteristics of fresh materials

The chemical composition and microbial population of fresh oat before ensiling are listed in Table 1. The DM content of the fresh oat was 22.29% FM. The chemical components including CP, WSC, NDF, and ADF were 7.80, 8.84, 59.80, and 39.62% DM, respectively. Moreover, the epiphytic LAB count of fresh oat in this study was 5.40 log_10_ cfu/g FM, and the counts for coliform bacteria, yeasts, and molds were 3.39, 4.13, and 5.32 log_10_ cfu/g FM, respectively.

**TABLE 1:** Chemical characteristics and microbial counts of fresh oat before fermentation.

	Items	Fresh oat
Chemical composition	DM (%FM)	22.29
	CP (%DM)	7.80
	WSC (%DM)	8.84
	NDF (%DM)	59.80
	ADF (%DM)	39.62
Microbial counts	LAB (log_10_ cfu/g FM)	5.40
	Coliform (log_10_ cfu/g FM)	3.99
	Mold (log_10_ cfu/g FM)	4.13
	Yeast (log_10_ cfu/g FM)	5.32

Data is the mean of three replicates. DM, dry matter; WSC, water-soluble carbohydrates; NDF, neutral detergent fiber; ADF, acid detergent fiber; LAB, lactic acid bacteria; FM, fresh matter; cfu, colony forming unit.

### 3.2. Fermentation characteristics of oat silage

As shown in Table 2, during the fermentation, the pH of all groups gradually decreased (from pH 4.38 to 4.22 for the CK group, from pH 4.15 to 4.01 for the LP694 group, from pH 4.07 to 3.95 for the LR753 group, and from pH 4.17 to 4.02 for the LPLR group) and on day 60, the LR753 group had a significantly lower (*p* < 0.05) pH, followed by LP694, LPLR, and CK groups. The AN gradually increased with time until day 14 of ensiling in all treatments. On day 60, the LR735 group had a significantly lower (*p* < 0.05) AN content (10.1 g kg^–1^ DM) followed by the LPLR (14.0 g kg^–1^ DM), LP694 (14.4 g kg^–1^ DM), and CK (15.3 g kg^–1^ DM) groups. There was no significant difference between the CK, LP694, and LPLR groups on day 60. During the whole fermentation process, the LA content of the four groups increased rapidly, and that of the LR753 group (60.95 g kg^–1^ DM) was significantly higher (*p* < 0.05) than that of the LPLR (54.61 g kg^–1^ DM), LP694 (48.27 g kg^–1^ DM), and CK (30.37 g kg^–1^ DM) groups on day 60. Meanwhile, the LA content of LR753, LP694, and LPLR on day 60 was numerically lower than that on day 14, while that of the CK was higher. The AA content of the four groups increased gradually with time. On day 60, the LAB-treated groups had a significantly higher (*p* < 0.05) AA content (LR753, 36.03 g kg^–1^ DM; LP694 33.73 g kg^–1^ DM; LPLR, 33.13 g kg^–1^ DM) than that of the CK group (19.83 g kg^–1^ DM). The AA content between the LAB-treated groups on day 60 was not significantly different. The PA content of the four groups gradually increased in time, and on day 60, the LR753 group had a significantly lower (*p* < 0.05) PA content (9.57 g kg^–1^ DM) than that of the LP694 (16.57 g kg^–1^ DM), LPLR (17.14 g kg^–1^ DM), and CK (18.03 g kg^–1^ DM) groups. The BA content first increased and then decreased in time in the LR753 and CK groups. On day 60, the LR753 group had a significantly lower (*p* < 0.05) BA content (1.33 g kg^–1^ DM), followed by the LPLR (1.98 g kg^–1^ DM), LP694 (2.67 g kg^–1^ DM), and CK (2.68 g kg^–1^ DM) groups.

**TABLE 2:** Fermentation characteristics of oat silages treated with and without lactic acid bacteria.

Items	Treatment	Ensiling days					SEM	Significance		
		1	3	7	14	60		T	D	T × D
pH	CK	4.38aA	4.30aA	4.25aB	4.24aB	4.22bC	0.004	***	***	***
	LP694	4.15bA	4.18bA	4.04cBC	4.05cB	4.01bC				
	LR753	4.07cB	4.04bA	3.97dC	3.93dD	3.95cD				
	LPLR	4.17bA	4.14bB	4.11bC	4.09bCD	4.02aD				
AN (g kg^–1^ DM)	CK	12.2aB	12.0abB	14.4aA	15.6aA	15.3aA	0.02	NS	***	***
	LP694	9.4cC	11.5abBC	13.2aA	14.3aA	14.4aA				
	LR753	9.7bcC	10.8bB	11.0bB	11.2bB	10.1bA				
	LPLR	1.02bD	1.23aC	13.3aBC	14.4aAB	14.0aA				
LA (g kg^–1^ DM)	CK	14.75bC	17.75cC	23.88bB	24.87bB	30.37cA	0.713	***	***	***
	LP694	20.95aD	27.63abCD	39.48abBC	62.43aA	48.27bAB				
	LR753	23.52aC	30.23aC	47.01aB	67.25aA	60.95aA				
	LPLR	21.29aC	23.90bC	40.16abB	64.40aA	54.61abA				
AA (g kg^–1^ DM)	CK	7.64bB	9.39bB	10.17bB	17.60cA	19.83bA	0.24	***	***	***
	LP694	8.91aD	10.67aD	19.08aC	24.87bB	33.73aA				
	LR753	9.42aD	10.40abD	21.90aC	32.07aB	36.03aA				
	LPLR	9.31aD	10.24abD	18.47aC	26.15bB	33.13aA				
PA (g kg^–1^ DM)	CK	5.25aE	9.36aD	10.59abC	13.10bB	18.03aA	0.151	***	***	**
	LP694	5.38aD	7.88bCD	9.87abBC	12.04bB	16.57abA				
	LR753	5.14aD	6.60cC	9.25bB	12.03bA	9.57bB				
	LPLR	5.28aE	7.19bcD	11.43aC	14.94aB	17.14aA				
BA (g kg^–1^ DM)	CK	2.87aA	1.84aC	1.49bC	2.31aBC	2.68aA	0.053	*	***	***
	LP694	1.49bB	1.53aB	1.71abB	2.65aA	2.67aA				
	LR753	1.68bAB	2.07aA	2.30aA	2.26aA	1.33cB				
	LPLR	1.41bC	1.54aBC	1.56abBC	2.46aA	1.98bB				

Data is the mean of three replicates. CK, sterilized water; LP694, *Lactobacillus plantarum* 694; LR753, Lactobacillus rhamnosus 753; LPLR, 50% LP694 + 50% LR753; AN, ammonia-N; LA, lactic acid; AA, acetic acid; PA, propionic acid; BA, butyric acid; DM, dry matter; NS, non-significant; SEM, standard error mean; T, treatment; D, ensiling day; T × D, interactive effect of treatment and ensiling days; *p < 0.05; **p < 0.01; ***p < 0.001. Capital letters indicate the significance within the same row, while small letters indicate the significance within the column.

### 3.3. Chemical composition and microbial counts of oat silage

As shown in Table 3, there was no significant difference in DM content between the groups throughout the fermentation, and on day 60, it ranged from 24.75 to 26.86%. During the fermentation, the WSC content of the four groups gradually decreased, and on day 60, the CK group had a significantly higher (*p* < 0.05) WSC content (5.33% DM) than that of the LAB-treated groups (LP694, 4.59% DM; LPLR 3.59% DM; and LR753, 3.15% DM). Meanwhile, there was no significant difference between the LAB-treated groups on 60 days. The NDF and ADF showed an irregular trend with fermentation time. On day 60, the LR753 group had a significantly lower (*p* < 0.05) NDF content (54.60% DM) compared to the LP674 (55.93% DM), LPLR (57.53% DM), and CK (56.33% DM) groups, while that of ADF content had no significant difference between groups. Meanwhile, the NDF (on day 3) and ADF (on 3 and day 14) contents of the CK group were comparable to the LAB-treated groups. All groups showed a significantly lower (*p* < 0.05) LAB count on day 60 compared to day 1. On day 60, the LR753 group had a substantially higher (*p* < 0.05) LAB count (6.31 log_10_ cfu/g FM) followed by the LP694 (5.26 log_10_ cfu/g FM), LPLR (5 log_10_ cfu/g FM), and CK (3.60 log_10_ cfu/g FM) groups. Other microbes (such as coliform bacteria, yeasts, and molds) were not detected in any group with prolonged fermentation time.

**TABLE 3:** Chemical characteristics and microbial counts of oat silages treated with or without lactic acid bacteria.

Items	Treatment	Ensiling days					SEM	Significance		
		1	3	7	14	60		T	D	T × D
DM (%)	CK	25.69aA	25.49aA	25.49aA	25.44aA	24.75aA	0.205	*	NS	NS
	LP694	26.10aA	25.50aA	27.00aA	24.53aA	26.06aA				
	LR753	26.92aA	27.20aA	27.38aA	26.24aA	26.85aA				
	LPLR	25.62aA	25.67aA	25.39aA	24.86aA	25.81aA				
WSC (%DM)	CK	8.40aA	6.57aB	5.86aB	5.45aB	5.33aB	0.082	***	***	NS
	LP694	8.89aA	5.88aB	4.96aB	5.40abB	4.59bB				
	LR753	8.77aA	4.88aB	4.73aBC	4.70bBC	3.15bC				
	LPLR	8.84aA	5.46aB	4.65aB	4.54bB	3.59bB				
NDF (%DM)	CK	56.53aA	55.13aA	56.13aA	56.53aA	56.33aA	0.133	***	***	**
	LP694	57.06aA	54.73aAB	54.80aAB	54.20bAB	55.93abB				
	LR753	56.60aA	55.60aAB	53.00bC	54.86abC	54.60bC				
	LPLR	57.06aAB	55.86aB	56.20aAB	54.46bC	57.53aA				
ADF (%DM)	CK	40.16abAB	39.52aAB	38.78abB	41.09aA	40.62aAB	0.143	***	*	NS
	LP694	40.57abAB	40.55aAB	39.96abB	39.43aB	41.82aA				
	LR753	38.94bAB	40.01aA	37.20bB	39.28aAB	39.73aAB				
	LPLR	41.04aA	40.45aA	40.73aA	40.18aA	42.23aA				
LAB (log_10_ cfu/g FM)	CK	7.51aA	6.82cB	6.86cB	6.24cC	3.63cD	0.094	***	***	***
	LP694	7.22bAB	7.34aA	7.26bA	6.99bB	5.26bC				
	LR753	7.20bB	7.31aAB	7.29bAB	7.52aA	6.31aC				
	LPLR	7.04bA	7.07bA	7.54aA	7.17bA	5.00bB				
Coliform (log_10_ cfu/g FM)	CK	6.30a	3.3	ND	ND	ND	-	-	-	-
	LP694	3.57c	ND	ND	ND	ND				
	LR753	4.02b	ND	ND	ND	ND				
	LPLR	3.20c	ND	ND	ND	ND				
Yeast (log_10_ cfu/g FM)	CK	<2.00	<2.00	<2.00	<2.00	ND	-	-	-	-
	LP694	<2.00	<2.00	ND	ND	ND				
	LR753	<2.00	<2.00	ND	ND	ND				
	LPLR	<2.00	<2.00	ND	ND	ND				

Data is the mean of three replicates. CK, sterilized water; LP694, *Lactobacillus plantarum* 694; LR753, *Lactobacillus rhamnosus* 753; LPLR, 50% LP694 + 50% LR753; DM, dry matter; WSC, water-soluble carbohydrates; NDF, neutral detergent fiber; ADF, acid detergent fiber; LAB, lactic acid bacteria; cfu, colony forming unit; FM, fresh matter; NS, non-significant; SEM, standard error mean; T, treatment; D, ensiling day; T × D, interactive effects of treatments and ensiling days; *p < 0.05; **p < 0.01; ***p < 0.001. Capital letters indicate the significance within the same row, and small letters indicate the significance within the column.

### 3.4. Bacterial community diversity in oat silage

The alpha diversity analysis of the bacterial community in fresh and ensiled samples is presented in Table 4. The good coverage value for all groups was above 0.99. The LR group had significantly (*p* < 0.05) lower Shannon and Simpson indexes than that of other groups throughout the fermentation. On day 60, except for the LP694 group, the LAB-treated groups showed lower Chao 1 and Ace indexes than the CK group. The exclusive OTUs of each group ranged from 10 to 531 throughout the fermentation (Supplementary Figure 1). The diversity of the bacterial central microbiota (consisting of 136 common OTUs) decreased after day 7 of fermentation and reached 110 on day 60. All groups except the LP694 group represented fewer OTUs while comparing day 1 to day 60 of fermentation. Principal coordinates analysis (PCoA) which was UniFrac-based showed a distinct clustering of the microbiota compositions for each group (Supplementary Figure 2). Principle coordinates 1 and 2 accounted for 78.11 and 6.01% of the total variance, respectively. The bacterial community in fresh material was clearly distinct from the ensiled oat.

**TABLE 4:** Influence of lactic acid bacteria inoculation on bacterial alpha diversity of oat silages.

Items	FM	Treatment	Ensiling days					SEM	Significance		
			1	3	7	14	60		T	D	T × D
Observed species	478	CK	181aA	175aA	232aA	149aA	137aA	11.028	NS	NS	*
		LP694	143aB	136aB	161aB	154aB	305aA				
		LR753	199aA	146aA	175aA	277aA	118aA				
		LPLR	139aB	169aAB	304aA	117aB	125aB				
Shannon	4.34	CK	2.54cD	3.49aC	4.19aA	3.90aAB	3.78abBC	0.034	***	***	***
		LP694	2.93bC	2.67bC	3.68abB	3.83aAB	4.22aA				
		LR753	3.16bA	2.59bB	3.11bA	3.13bA	2.52cB				
		LPLR	3.82aAB	3.57aBC	4.10aA	3.17bC	3.68bABC				
Simpson	0.91	CK	0.60aC	0.81aB	0.92aA	0.90aA	0.86aA	0.005	***	***	***
		LP694	0.73cB	0.64bC	0.85aA	0.89aA	0.92aA				
		LR753	0.78bB	0.63bA	0.73bA	0.73bA	0.64bB				
		LPLR	0.89aA	0.83aA	0.88aA	0.76bB	0.87aA				
Chao 1	537	CK	198aA	193aA	252aA	165abA	157bA	12.473	NS	NS	*
		LP694	155aB	149aB	176aB	173abB	339aA				
		LR753	222aA	185aA	203aA	334aA	140bA				
		LPLR	149aB	187aAB	341aA	127bB	139bB				
Ace	563	CK	206aA	200aA	263aA	173abA	159bA	12.782	NS	NS	NS
		LP694	162aB	156aB	184aB	180abB	349aA				
		LR753	231aA	180aA	209aA	336aA	147bA				
		LPLR	152aA	192aAB	346aA	137bA	143bA				
Coverage	0.99	CK	0.99	0.99	0.99	0.99	0.99	-	NS	NS	NS
		LP694	1	1	0.99	0.99	0.99				
		LR753	1	0.99	0.99	0.99	0.99				
		LPLR	1	0.99	0.99	1	1				

Data is the mean of four replicates. CK, sterilized water; LP694, *Lactobacillus plantarum* 694; LR753, *Lactobacillus rhamnosus* 753; LPLR, 50% LP694 + 50% LR753; FM, fresh oat material; NS, non-significant; SEM, standard error mean; T, treatment; D, ensiling day; T × D, interactive effects of treatments and ensiling days; *p < 0.05; ***p < 0.001. Capital letters indicate the significance within the same row, while small letters indicate the significance within the column.

**FIGURE 1:**
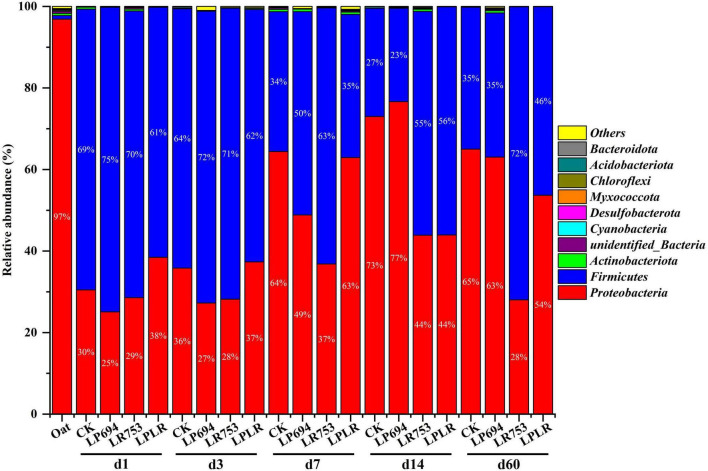
Relative abundance of bacteria at phylum level for oat silages at different days of fermentation with and without lactic acid bacterial inoculation. CK, sterilized water; LP694, *Lactobacillus plantarum* 694; LR753, *Lactobacillus rhamnosus* 753; LPLR, 50% LP694 + 50% LR753; d, ensiling days; OAT.F, fresh oat.

**FIGURE 2:**
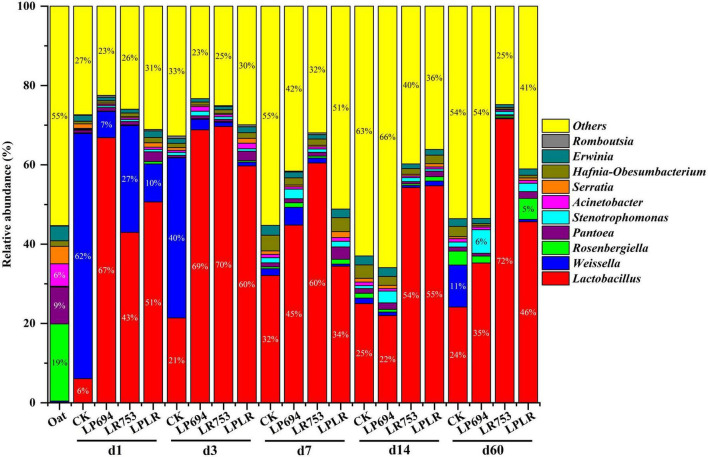
Relative abundance of bacteria at the genus level for oat silages at different days of ensiling with and without lactic acid bacterial inoculation. CK, sterilized water; LP694, *Lactobacillus plantarum* 694; LR753, *Lactobacillus rhamnosus* 753; LPLR, 50% LP694 + 50% LR753; d, ensiling days; OAT.F, fresh oat.

The microbial community composition of oat silages was represented by two major phyla: *Proteobacteria* and *Firmicutes* (Figure 1). *Proteobacteria* was the dominant phyla in fresh oat accounting for a total relative abundance of 97%. After ensiling, *Firmicutes* became the dominant phyla on day 3 of fermentation (CK, 64%; LP694, 72%; LR753, 71%; and LPLR, 62%). However, *Proteobacteria* dominated again in all groups (CK, 65%; LP694, 63%; and LPLR, 54%) except for the LR753 group (*Firmicutes*, 72%) on day 60. The *Rosenbergiella* (19%) and *Pantoea* (9%) were the dominant genera in the fresh oat followed by *Acinetobacter* (6%) (Figure 2). Notably, the relative abundance of the genus *Lactobacillus* was less than 1% in the fresh oat, but it gradually became the most prevalent genus in all groups with fermentation time. The relative abundance of *Lactobacillus* increased in the CK group (from 6% on day 1 to 32% on day 7) and then slightly decreased with further fermentation time (24% on day 60). The relative abundance of *Lactobacillus* increased rapidly on day 3 in the LAB-treated groups (LP694, 69%; LR753, 70%; and LPLR, 60%). However, its relative abundance decreased significantly in LP694 (35%) and LPLR (46%) groups on day 60, while that of the LR753 group showed a higher relative abundance of 72%. The relative abundance of *Weissella* decreased significantly in the CK group (from 62% on day 1 to 11% on day 60), while its relative abundance continuously decreased in the LAB-treated groups from 27% on day 1 to 0.03% on day 60. Moreover, *Pantoea*, *Rosenbergiella*, *Erwinia*, *Stenotrophomonas*, and *Acinetobacter* were the minor genera in all groups accounting for less than 10% of the total genera throughout the fermentation period.

**FIGURE 3:**
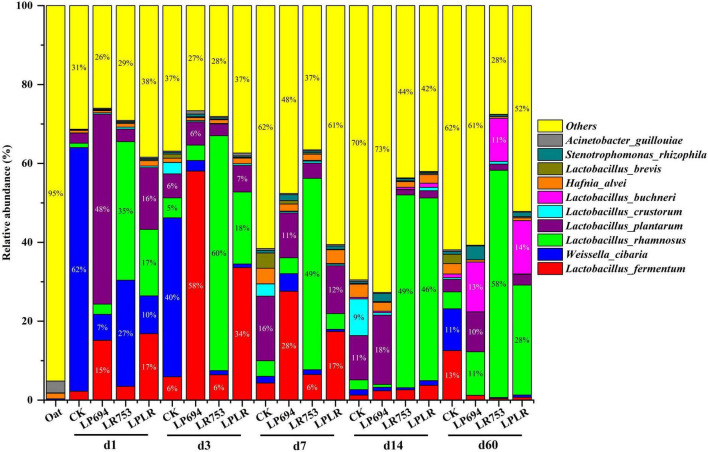
Relative abundance of bacteria at the species level for oat silages at different days of ensiling with and without lactic acid bacterial inoculation. CK, sterilized water; LP694, *Lactobacillus plantarum* 694; LR753, *Lactobacillus rhamnosus* 753; LPLR, 50% LP694 + 50% LR753; d, ensiling days; OAT.F, fresh oat.

The dynamic changes in the bacterial community composition and succession at the species level in oat silages are shown in Figure 3. The *Acinetobacter guillouiae* (3%) and *Hafnia alvei* (1.4%) were the most prevalent species in the fresh oat. The *Weissella cibaria* was the abundant specie in the CK group during the early phase of fermentation (62% on day 1 and 40% on day 3), however, *Lactobacillus plantarum* became the prevalent specie in the later phase of fermentation (16% on day 7 and 11% on day 14). As expected, the *Lactobacillus* species such as *Lactobacillus rhamnosus, Lactobacillus plantarum*, and *Lactobacillus fermentum* became the prevalent species in the LAB-treated groups with fermentation time. The *Lactobacillus plantarum* (48% on day 1) and *Lactobacillus fermentum* (58% on day 3) were abundant species in the LP694 group during the early phase of fermentation, and on day 60 *Lactobacillus rhamnosus* (11%), *Lactobacillus buchneri* (13%), and *Lactobacillus plantarum* (10%) became the dominant species. The LR753 had a significantly greater relative abundance of *Lactobacillus rhamnosus* throughout the fermentation (35% on day 1, 49% on day 7, and 58% on day 60). The *Lactobacillus fermentum* (34% on day 3), *Lactobacillus rhamnosus* (18% on day 3), and *Lactobacillus plantarum* (12% on day 7) species were dominated during the initial phase of fermentation in the LPLR silage; however, *Lactobacillus rhamnosus* (28%) and *Lactobacillus buchneri* (14%) species were the abundant species on day 60. These results suggest that the microbial inoculants have a significant influence on the bacterial community of the dynamically ensiled oat.

### 3.5. Correlation analysis

**FIGURE 4:**
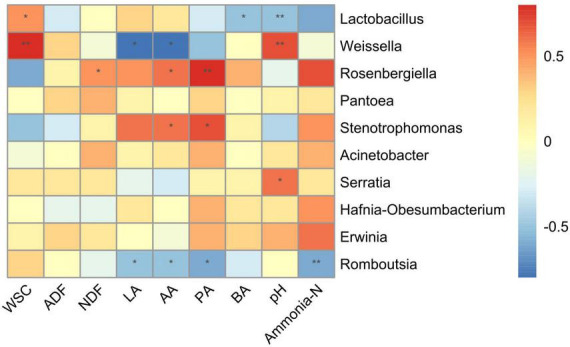
Correlation analysis between the bacterial community at genus level and fermentation products. WSC, water-soluble carbohydrates; NDF, neutral detergent fiber; ADF, acid detergent fiber; LA, lactic acid; AA, acetic acid; PA, propionic acid; BA, butyric acid. The corresponding value of heatmap is the Spearman correlation coefficient r (−1 to 1), a value above 0 indicates a positive correlation (red), a value below 0 shows a negative correlation (red), *p < 0.05 and ^**^p < 0.01.

The correlation analysis between microbiota at the genus level and fermentation products was conducted to investigate the effects of the microbial community on the fermentation quality (Figure 4). The *Lactobacillus* was positively correlated (*R* = 0.6, *p* < 0.05) with WSC, while negatively correlated with pH (*R* = −0.5, *p* < 0.05), and BA (*R* = −0.5, *p* < 0.01). The *Weissella* showed a positive correlation with WSC (*R* = 0.8, *p* < 0.01) and pH (*R* = 0.7, *p* < 0.01), while had a negative correlation with LA (*R* = −0.7, *p* < 0.05) and AA (*R* = −0.7, *p* < 0.05). The *Rosenbergiella* was positively correlated with PA (*R* = 0.7, *p* < 0.01), AA (*P* = 0.6, *p* < 0.05), and NDF (*R* = 0.5, *p* < 0.05), while *Stenotrophomonas* was positively correlated with PA (*R* = 0.6, *p* < 0.05) and AA (*R* = 0.6, *p* < 0.05). The *Serratia* had a positive correlation (*R* = 0.6, *p* < 0.05) with pH, while *Romboutsia* showed negative correlations with LA (*R* = −0.5, *p* < 0.05), AA (*R* = −0.5, *p* < 0.05), PA (*R* = −0.6, *p* < 0.05), and ammonia-N (*R* = −0.6, *p* < 0.05).

## 4. Discussion

### 4.1. Silage quality of oat with or without lactic acid bacteria inoculation

The silage quality is greatly dependent on the substrates present in fresh material and is mainly driven by microbial activity (Ni et al., 2017). The pH of silage is an important indicator for silage fermentation (Muck et al., 2018). In the current study, the pH of all groups decreased with fermentation time; however, rapid acidification was seen in the LAB-treated groups. This might be related to the beneficial effects of the exogenous application of LAB which utilize WSC to produce more LA leading to a reduction in pH (Yan et al., 2019). The pH of the LAB-treated groups was below 4.2, and the LR753 group had the lowest pH of 3.95 on day 60 among all groups. It is acknowledged that a pH of 4.2 is a benchmark for well-preserved silage (Ni et al., 2018). Contrarily, the CK group had a higher pH compared to the LAB-treated groups which might be attributed to its lower LA content. The AA is a major end product of the heterofermentative LAB and a crucial substance for aerobic stability (McDonald et al., 1991). The AA concentration increased with fermentation time, and the LAB-treated groups had greater AA concentrations compared to the CK group on day 60. The increase of AA concentrations during fermentation may be related to yeast and some LAB (*Lactobacillus hilgardii* and *Lactobacillus buchneri*), as they can metabolize LA into AA under certain conditions (Guan et al., 2018; Bai et al., 2022). The PA concentration of 1–10 g kg^–1^ DM is considered a standard in silage (Agarussi et al., 2019). Meanwhile, only the LR753 group met the PA general standard in the current study highlighting that the LR753 strain can restrict the growth of PA-producing microflora. The BA is usually produced by undesirable bacteria which decompose the CP resulting in nutrient loss (Kung et al., 2018). The BA concentrations in all groups were significantly higher in the early phase of fermentation compared to the later phase of fermentation which might be related to the greater relative abundance of pathogenic or spoilage microorganisms during early ensiling. However, the LR753 group had a lower BA concentration than that of other groups which might be related to its greater acidification environment in which the growths of pathogenic or spoilage microorganisms such as *Stenotrophomonas rhizophila* were restricted.

The LAB count was higher in the inoculated groups after day 1 of ensiling than in the CK silage, which was in line with the results of decreased pH values in inoculated silages which inhibited the growth of undesirable microbes such as coliform, yeasts, and molds. Meanwhile, the LAB count decreased with prolonged ensiling time in all groups which might be attributed to a decrease in the substrate such as WSC concentrations. However, the other microbes such as coliform, yeasts, and molds were not detected after day 60 of ensiling, which might be attributed to the acidic environment. The DM content is an important index of the nutritional preservation of forages (Hu et al., 2009). The DM content was marginally influenced by the fermentation or LAB inoculation in the present study and consistent with the work of Liu et al. (2019). Perhaps, it could have occurred because the low pH in all the silages was acidic enough to inhibit nutrient degradation during ensiling. Meanwhile, a previous study reported that *Lactobacillus plantarum*, or a combination of LAB inoculation, increased DM concentration in the alfalfa and other legumes, and temperate and tropical grasses (Oliveira et al., 2017). The level of AN is an indicator of CP degradation in the silage (Wang et al., 2019). It is worth noting that the AN was significantly lower in the LAB-treated groups than the CK group, highlighting that undesirable proteolytic bacteria were inhibited effectively in the LAB-treated silages. This could be attributed to the sharp pH decline in such silages which restricted the growth of undesirable microorganisms (Guan et al., 2018). The ADF and NDF concentrations were decreased in the LR753 group than that of other groups on day 60. This could be exemplified by the more digestible cell wall fractions hydrolyzed by enzymes and acids during silage fermentation (Yan et al., 2019). The WSC contents were decreased in all groups after fermentation suggesting that it was utilized and broken down into organic acids by LAB during fermentation (Xia et al., 2022). The decreasing trend of WSC contents of all groups was consistent with the pH value.

### 4.2. Microbial community of oat silage with or without lactic acid bacteria inoculation

The microbial DNA sample coverage in this study was nearly one, suggesting that most of the bacteria in the samples were represented by 16S rDNA sequences. The alpha diversity was significantly affected by fermentation time. The different Shannon and Chao1 indices among groups on day 1 and day 60 highlighted that the richness and evenness of the bacterial communities were not stable at the initial and terminal stages of fermentation when LAB was inoculated. The OUTs and PCoA analysis suggested that LAB inoculation affected the microbial composition, and all groups had different bacterial populations after fermentation in agreement with previous reports (Guan et al., 2021; Zi et al., 2021). This could be attributed to the changes in the level of some taxonomic groups that were offset by opposite changes in other groups (Hartmann and Widmer, 2006). The samples treated with LAB were separated suggesting that exogenous microbiota could change the microbial community succession in oat silage. Moreover, the samples on different fermentation times were evidently separated highlighting that microbial community changed with fermentation time within the groups. However, it was quite fascinating to know that the microbiota of the LR753 group on day 3 and day 60 were clustered together suggesting that LR753 strain inoculation could promote a homo-fermentative process and the resultant silage thus remained stable in an acidic environment.

The bacterial community is correlated with the fermentation quality because the fermentation process is highly dependent on the interactions of multiple bacteria (Ni et al., 2017). The *Proteobacteria* and *Firmicutes* were the dominant phyla in all groups after fermentation consistent with previous work (Liu et al., 2019). This might be attributed to the acidification and anaerobic conditions which were conducive to the growth of these two phyla. The *Firmicutes* are vital acid hydrolytic microbes under anaerobic conditions, which could produce numerous extracellular enzymes (Wang S. et al., 2020). The LR753 group had a substantially greater relative abundance of *Firmicutes* and a lesser relative abundance of *Proteobacteria* compared to other LAB-treated groups on day 60 highlighting that microbial composition structure changed significantly when *the* LR753 strain was inoculated. The possible reason for this could be the rapid and more acidification (pH < 4) in the LR753 group which was conducive to the growth of *Firmicutes* compared to *Proteobacteria* (Keshri et al., 2018). The *Rosenbergiella* and *Pantoea* were the most prevalent genera in the fresh oat. Generally, the *Rosenbergiella* and *Pantoea* compete with LAB for sugars, and their presence in silage is considered undesirable (Jiang et al., 2020). However, the growths of *Rosenbergiella* and *Pantoea* were inhibited significantly after fermentation which might be related to the reduction of pH in all groups. The succession of bacteria is a dynamic process that varies in the different phases of fermentation. It has been reported that the *Lactobacillus* genus dominates the fermentation process under anaerobic conditions and could grow vigorously during the fermentation stage due to its stronger acid resistance (Li et al., 2022). The *Lactobacillus* was the dominant genus in the LAB-treated groups throughout the fermentation consistent with previous research (Liu et al., 2019). This might be because of the anaerobic conditions that facilitate the growth of LAB strains, which could produce LA to enhance their competitiveness by inhibiting other background bacteria (Muck et al., 2018). Meanwhile, *Weissella* was the dominant genus during the early phase of fermentation in the CK group. The *Weissella* is usually regarded as an early colonizer (Dellaglio and Torriani, 1986), and surpassed by acid-resistant *Lactobacillus* because of pH decline as fermentation starts (Graf et al., 2016). Similarly, in the current study, the relative abundance of *Weissella* in the CK group decreased significantly in the later phase of fermentation. The possible reason for this outcome might be the acidic environment (lower pH), which was not suitable for the growth of *Weissella*.

The *Acinetobacter guillouiae* and *Hafnia alvei* were the most dominant species in fresh oat. The *Weissella cibaria* was abundant specie in the CK group during the early phase of fermentation but it was replaced by *Lactobacillus plantarum* specie in the later phase of fermentation. This might be due to the greater pH value during the early phase of fermentation which was conducive to the growth of *Weissella cibaria*, while the lower pH value at the later phase of fermentation was favorable for the growth of *Lactobacillus plantarum* as its growth depends on the pH. Therefore, the lower relative abundance of *Lactobacillus* species in the CK group may explain its poor fermentation quality exemplified by the greater pH value, lower LA content, and greater AN concentration. As expected, the LAB-treated groups had greater relative abundances of *Lactobacillus* species throughout the fermentation, and the major species were *Lactobacillus rhamnosus*, *Lactobacillus plantarum*, and *Lactobacillus buchneri*. Similarly, studies have established the inoculation of LAB increased the relative abundances of *Lactobacillus* species in the silages (Yan et al., 2019; Xia et al., 2022). The LR753 group had a greater relative abundance of *Lactobacillus rhamnosus* on day 60 than that of other groups which may explain its higher LA concentration and lower pH value and AN content. It was quite fascinating to know that the relative abundance of *Lactobacillus plantarum* was significantly lower than that of *Lactobacillus rhamnosus* in the LPLR group on day 60. This might be attributed to the greater acidic tolerance and the substrates competitive abilities of *Lactobacillus rhamnosus* compared to *Lactobacillus plantarum*. Moreover, previous studies have reported that the relative abundance of homofermentative LAB decreased, while the relative abundance of heterofermentative LAB increased in the later or stable phase of fermentation (Zhou et al., 2016; He et al., 2019). In the current study, the substantial relative abundances of *Lactobacillus buchneri* were found in LAB-treated groups on day 60 but failed to dominate in these groups. It has been well established that heterofermentative LAB produces the AA by utilizing WSC as a substrate which is a key substance for silage aerobic stability (Muck et al., 2018). Therefore, the presence of heterofermentative *Lactobacillus buchneri* highlights that it could promote the aerobic stability of silage by producing more AA contents.

### 4.3. Correlation between fermentation products and microbial community

The silage process is largely related to microbial communities and biochemical reactions, and the fermentation of silage is largely dependent on the microbial community and a series of end products (Zhao et al., 2021; Li et al., 2022). The present study showed a significant correlation between the fermentation products and the bacterial community which was similar to the results of McAllister et al. (2018). The genus *Lactobacillus* had a positive correlation with WSC and LA and a negative correlation with pH and AN. Similar interactions were found by Xia et al. (2022), who reported that *Lactobacillus* promotes the accumulation of LA, and inhibits the production of AN by decreasing pH leading to quality fermentation. The *Rosenbergiella* was positively correlated with PA, AA, and NDF, highlighting that it is involved in the degradation of the plant cell wall and can increase NDF contents. Recently, *Rosenbergiella* has been described as a new genus of *Enterobacteria*, which are Gram-negative rods, facultative anaerobes, and can ferment lactose to acids (Lenaerts et al., 2014). However, the role of *Rosenbergiella* in silage fermentation is not well studied yet. Moreover, *Romboutsia* was negatively correlated with organic acids and AN suggesting that it obstructs the production of organic acids and proteolysis (Li et al., 2020). The *Weissella* and *Serratia* were positively correlated with pH, suggesting that these are involved with silage corruption. Overall, the bacterial community substantially influenced the silage quality by affecting the pH, organic acids, and AN concentrations, and their effects were bidirectional.

## 5. Conclusion

The LAB addition could improve the silage quality of oat to different extents, but the oat silage treated with LR753 had better fermentation quality than that of other groups. The inoculation of LR753 could enhance the relative abundance of desirable *Latobacillus*, thereby increasing the LA content, and decreasing the pH value, AN, NDF, and ADF contents to improve the fermentation quality of oat silage. Overall, LR753 can effectively preserve nutrients in silage and improve fermentation quality which provides the theoretical support and guidance for future oat silage production in Southwest China that may further assist the current shifts toward sustainable and high-efficiency agricultural production systems.

## Data availability statement

The datasets presented in this study can be found in online repositories. The names of the repository/repositories and accession number(s) can be found in the article/Supplementary material.

## Author contributions

MT: writing—original draft preparation, methodology, software, and formal analysis. JL: methodology and resources. YX, CC, YZ, and LZ: investigation and data curation. TW: writing—review and editing, and visualization. HL and YH: validation and formal analysis. XW: resources and validation. YY: project administration, funding acquisition, supervision, and investigation. All authors contributed to the article and approved the submitted version.

## Footnotes

1
http://cutadapt.readthedocs.io/en/stable/

2
http://www.drive5.com/uparse/

3
http://www.arb-silva.de/

## Supplementary material

Supplementary Figure 1 Venn diagram depicting the common or unique bacterial OTU of oat silages at different days of fermentation: **(A)** 1 day; **(B)** 3 days; **(C)** 7 days; **(D)** 14 days; and **(E)** 60 days. CK, sterilized water; LP, *Lactobacillus plantarum* 694; LR, *Lactobacillus rhamnosus* 753; LPLR, 50% LP + 50% LR; d, ensiling days.

Supplementary Figure 2 Principal component analysis of bacterial community during ensiling at different days of fermentation with and without lactic acid bacterial inoculation. CK, sterilized water; LP, *Lactobacillus plantarum* 694; LR, *Lactobacillus rhamnosus* 753; LPLR, 50% LP + 50% LR; d, ensiling days; OAT.F, fresh oat.

## References

[B1] Agarussi M. C. N. Pereira O. G. Da Silva V. P. Leandro E. S. Ribeiro K. G. Santos S. A. (2019). Fermentative profile and lactic acid bacterial dynamics in non-wilted and wilted alfalfa silage in tropical conditions. *Mol. Biol. Rep.* 46 451–460. 10.1007/s11033-018-4494-z 30443821

[B2] Andrzejewska J. Contreras-Govea F. E. Pastuszka A. Kotwica K. Albrecht K. A. (2019). Performance of oat (*Avena sativa* L.) sown in late summer for autumn forage production in Central Europe. *Grass Forage Sci.* 74 97–103. 10.1111/gfs.12400

[B3] Association of Official Analytical Chemists [AOAC] (1990). *Official methods of analysis.* Arlington, VA: Association of Official Analytical Chemists.

[B4] Bai J. Ding Z. Su R. Wang M. Cheng M. Xie D. (2022). Storage temperature is more effective than lactic acid bacteria inoculations in manipulating fermentation and bacterial community diversity, co-occurrence and functionality of the whole-plant corn silage. *Microbiol. Spectr.* 10:e0010122. 10.1128/spectrum.00101-22 35343767 PMC9045155

[B5] Broderick G. Kang J. (1980). Automated simultaneous determination of ammonia and total amino acids in ruminal fluid and in vitro media1. *J. Dairy Sci.* 63 64–75. 10.3168/jds.S0022-0302(80)82888-8 7372898

[B6] Cheng Q. Chen L. Chen Y. Li P. Chen C. (2022). Effects of LAB inoculants on the fermentation quality, chemical composition, and bacterial community of oat silage on the Qinghai-Tibetan Plateau. *Microorganisms* 10:787. 10.3390/microorganisms10040787 35456837 PMC9026496

[B7] Dellaglio F. Torriani S. (1986). DNA-DNA homology, physiological characteristics and distribution of lactic acid bacteria isolated from maize silage. *J. Appl. Bacteriol.* 60 83–92. 10.1111/j.1365-2672.1986.tb03363.x

[B8] Diao X. (2017). Production and genetic improvement of minor cereals in China. *Crop J.* 5 103–114.

[B9] Edgar R. C. (2013). UPARSE: Highly accurate OTU sequences from microbial amplicon reads. *Nat. Methods* 10 996–998. 10.1016/j.cj.2016.06.004 23955772

[B10] Gebrehanna M. Gordon R. Madani A. Vanderzaag A. Wood J. (2014). Silage effluent management: A review. *J. Environ. Manag.* 143 113–122. 10.1016/j.jenvman.2014.04.012 24905641

[B11] Graf K. Ulrich A. Idler C. Klocke M. (2016). Bacterial community dynamics during ensiling of perennial ryegrass at two compaction levels monitored by terminal restriction fragment length polymorphism. *J. Appl. Microbiol.* 120 1479–1491. 10.1111/jam.13114 26923533

[B12] Guan H. Ke W. Yan Y. Shuai Y. Li X. Ran Q. (2020). Screening of natural lactic acid bacteria with potential effect on silage fermentation, aerobic stability and aflatoxin B1 in hot and humid area. *J. Appl. Microbiol.* 128 1301–1311. 10.1111/jam.14570 31898381

[B13] Guan H. Ran Q. Li H. Zhang X. (2021). Succession of microbial communities of corn silage inoculated with heterofermentative lactic acid bacteria from ensiling to aerobic exposure. *Fermentation* 7:258. 10.3390/fermentation7040258

[B14] Guan H. Yan Y. Li X. Li X. Shuai Y. Feng G. (2018). Microbial communities and natural fermentation of corn silages prepared with farm bunker-silo in Southwest China. *Bioresour. Technol.* 265 282–290. 10.1016/j.biortech.2018.06.018 29908496

[B15] Haas B. J. Gevers D. Earl A. M. Feldgarden M. Ward D. V. Giannoukos G. (2011). Chimeric 16S rRNA sequence formation and detection in Sanger and 454-pyrosequenced PCR amplicons. *Genome Res.* 21 494–504. 10.1101/gr.112730.110 21212162 PMC3044863

[B16] Hartmann M. Widmer F. (2006). Community structure analyses are more sensitive to differences in soil bacterial communities than anonymous diversity indices. *Appl. Environ. Microbiol.* 72 7804–7812. 10.1128/AEM.01464-06 17041161 PMC1694274

[B17] He L. Wang C. Xing Y. Zhou W. Pian R. Chen X. (2020). Ensiling characteristics, proteolysis and bacterial community of high-moisture corn stalk and stylo silage prepared with Bauhinia variegate flower. *Bioresour. Technol.* 296:122336. 10.1016/j.biortech.2019.122336 31704603

[B18] He L. Wang C. Xing Y. Zhou W. Pian R. Yang F. (2019). Dynamics of proteolysis, protease activity and bacterial community of Neolamarckia cadamba leaves silage and the effects of formic acid and *Lactobacillus farciminis*. *Bioresour. Technol.* 294:122127. 10.1016/j.biortech.2019.122127 31525585

[B19] Hu W. Schmidt R. J. Mcdonell E. E. Klingerman C. M. Kung L. (2009). The effect of *Lactobacillus buchneri* 40788 or *Lactobacillus plantarum* MTD-1 on the fermentation and aerobic stability of corn silages ensiled at two dry matter contents. *J. Dairy Sci.* 92 3907–3914. 10.3168/jds.2008-1788 19620673

[B20] Jiang F.-G. Cheng H.-J. Liu D. Wei C. An W.-J. Wang Y.-F. (2020). Treatment of whole-plant corn silage with lactic acid bacteria and organic acid enhances quality by elevating acid content, reducing pH, and inhibiting undesirable microorganisms. *Front. Microbiol.* 11:593088. 10.3389/fmicb.2020.593088 33343533 PMC7746776

[B21] Keshri J. Chen Y. Pinto R. Kroupitski Y. Weinberg Z. G. (2018). Microbiome dynamics during ensiling of corn with and without *Lactobacillus plantarum* inoculant. *Appl. Microbiol. Biotechnol.* 102 4025–4037. 10.1007/s00253-018-8903-y 29536147

[B22] Kung L. Jr. Shaver R. Grant R. Schmidt R. (2018). Silage review: Interpretation of chemical, microbial, and organoleptic components of silages. *J. Dairy Sci.* 101 4020–4033. 10.3168/jds.2017-13909 29685275

[B23] Lenaerts M. Álvarez-Pérez S. De Vega C. Van Assche A. Johnson S. D. Willems K. A. (2014). *Rosenbergiella australoborealis* sp. nov., *Rosenbergiella collisarenosi* sp. nov. and *Rosenbergiella epipactidis* sp. nov., three novel bacterial species isolated from floral nectar. *Syst. Appl. Microbiol.* 37 402–411. 10.1016/j.syapm.2014.03.002 24794950

[B24] Li P. Lu Y. Zhao M. Chen L. Zhang C. Cheng Q. (2022). Effects of phenyllactic acid, lactic acid bacteria, and their mixture on fermentation characteristics and microbial community composition of timothy silage. *Front. Microbiol.* 12:743433. 10.3389/fmicb.2021.743433 34975781 PMC8716789

[B25] Li R. Jiang D. Zheng M. Tian P. Zheng M. Xu C. (2020). Microbial community dynamics during alfalfa silage with or without clostridial fermentation. *Sci. Rep.* 10:17782. 10.1038/s41598-020-74958-1 33082504 PMC7576192

[B26] Liu B. Huan H. Gu H. Xu N. Shen Q. Ding C. (2019). Dynamics of a microbial community during ensiling and upon aerobic exposure in lactic acid bacteria inoculation-treated and untreated barley silages. *Bioresour. Technol.* 273 212–219. 10.1016/j.biortech.2018.10.041 30447622

[B27] Martin M. (2011). Cutadapt removes adapter sequences from high-throughput sequencing reads. *EMBnet. J* 17 10–12. 10.14806/ej.17.1.200

[B28] McAllister T. Dunière L. Drouin P. Xu S. Wang Y. Munns K. (2018). Silage review: Using molecular approaches to define the microbial ecology of silage. *J. Dairy Sci.* 101 4060–4074. 10.3168/jds.2017-13704 29685277

[B29] McDonald P. Henderson A. Heron S. J. E. (1991). *The biochemistry of silage.* London: Chalcombe publications.

[B30] Muck R. Nadeau E. Mcallister T. Contreras-Govea F. Santos M. Kung L. Jr. (2018). Silage review: Recent advances and future uses of silage additives. *J. Dairy Sci.* 101 3980–4000. 10.3168/jds.2017-13839 29685273

[B31] Ni K. Wang F. Zhu B. Yang J. Zhou G. Pan Y. (2017). Effects of lactic acid bacteria and molasses additives on the microbial community and fermentation quality of soybean silage. *Bioresour. Technol.* 238 706–715. 10.1016/j.biortech.2017.04.055 28501002

[B32] Ni K. Zhao J. Zhu B. Su R. Pan Y. Ma J. (2018). Assessing the fermentation quality and microbial community of the mixed silage of forage soybean with crop corn or sorghum. *Bioresour. Technol.* 265 563–567. 10.1016/j.biortech.2018.05.097 29861298

[B33] Oliveira A. S. Weinberg Z. G. Ogunade I. M. Cervantes A. A. Arriola K. G. Jiang Y. (2017). Meta-analysis of effects of inoculation with homofermentative and facultative heterofermentative lactic acid bacteria on silage fermentation, aerobic stability, and the performance of dairy cows. *J. Dairy Sci.* 100 4587–4603. 10.3168/jds.2016-11815 28342607

[B34] Reynolds S. G. Suttie J. M. (2004). *Fodder oats: A world overview.* Princeton, NJ: Citeseer.

[B35] Rognes T. Flouri T. Nichols B. Quince C. Mahé F. (2016). VSEARCH: A versatile open source tool for metagenomics. *PeerJ* 4:e2584. 10.7717/peerj.2584 27781170 PMC5075697

[B36] Van Soest P. V. Robertson J. Lewis B. (1991). Methods for dietary fiber, neutral detergent fiber, and nonstarch polysaccharides in relation to animal nutrition. *J. Dairy Sci.* 74 3583–3597. 10.3168/jds.S0022-0302(91)78551-2 1660498

[B37] Wang S. Zhao J. Dong Z. Li J. Kaka N. A. Shao T. (2020). Sequencing and microbiota transplantation to determine the role of microbiota on the fermentation type of oat silage. *Bioresour. Technol.* 309:123371. 10.1016/j.biortech.2020.123371 32305853

[B38] Wang T. Teng K. Cao Y. Shi W. Xuan Z. Zhou J. (2020). Effects of *Lactobacillus hilgardii* 60TS-2, with or without homofermentative *Lactobacillus plantarum* B90, on the aerobic stability, fermentation quality and microbial community dynamics in sugarcane top silage. *Bioresour. Technol.* 312:123600. 10.1016/j.biortech.2020.123600 32531735

[B39] Wang Y. He L. Xing Y. Zhou W. Pian R. Yang F. (2019). Bacterial diversity and fermentation quality of Moringa oleifera leaves silage prepared with lactic acid bacteria inoculants and stored at different temperatures. *Bioresour. Technol.* 284 349–358. 10.1016/j.biortech.2019.03.139 30954903

[B40] Xia T. Wang T. Sun J. Shi W. Liu Y. Huang F. (2022). Modulation of fermentation quality and metabolome in co-ensiling of *Sesbania cannabina* and sweet sorghum by lactic acid bacterial inoculants. *Front. Microbiol.* 13:851271. 10.3389/fmicb.2022.851271 35401441 PMC8988063

[B41] Xu J. Zhang K. Lin Y. Li M. Wang X. Yu Q. (2022). Effect of cellulase and lactic acid bacteria on the fermentation quality, carbohydrate conversion, and microbial community of ensiling oat with different moisture contents. *Front. Microbiol.* 13:1013258. 10.3389/fmicb.2022.1013258 36274697 PMC9581316

[B42] Yan Y. Li X. Guan H. Huang L. Ma X. Peng Y. (2019). Microbial community and fermentation characteristic of Italian ryegrass silage prepared with corn stover and lactic acid bacteria. *Bioresour. Technol.* 279 166–173. 10.1016/j.biortech.2019.01.107 30721817

[B43] Yang F. Wang Y. Zhao S. Feng C. Fan X. (2022). Dynamics of the fermentation products, residual non-structural carbohydrates, and bacterial communities of wilted and non-wilted alfalfa silage with and without *Lactobacillus plantarum* inoculation. *Front. Microbiol.* 12:824229. 10.3389/fmicb.2021.824229 35087507 PMC8788936

[B44] Zeng T. Li X. Guan H. Yang W. Liu W. Liu J. (2020). Dynamic microbial diversity and fermentation quality of the mixed silage of corn and soybean grown in strip intercropping system. *Bioresour. Technol.* 313:123655. 10.1016/j.biortech.2020.123655 32559709

[B45] Zhang M. Tan Z. Wang X. Cui M. Wang Y. Jiao Z. (2018). Impact inoculum dosage of lactic acid bacteria on oat and wheat silage fermentation at ambient and low temperatures. *Crop Pasture Sci.* 69 1225–1236. 10.1071/CP17250

[B46] Zhang Q. Yu Z. Wang X. (2015). Isolating and evaluating lactic acid bacteria strains with or without sucrose for effectiveness of silage fermentation. *Grassland Sci.* 61 167–176. 10.1111/grs.12097

[B47] Zhao S. Yang F. Wang Y. Fan X. Feng C. Wang Y. (2021). Dynamics of fermentation parameters and bacterial community in high-moisture alfalfa silage with or without lactic acid bacteria. *Microorganisms* 9:1225. 10.3390/microorganisms9061225 34200084 PMC8226466

[B48] Zhou Y. Drouin P. Lafrenière C. (2016). Effect of temperature (5–25^°^ C) on epiphytic lactic acid bacteria populations and fermentation of whole-plant corn silage. *J. Appl. Microbiol.* 121 657–671. 10.1111/jam.13198 27271320

[B49] Zi X. Li M. Chen Y. Lv R. Zhou H. Tang J. (2021). Effects of citric acid and *Lactobacillus plantarum* on silage quality and bacterial diversity of king grass silage. *Front. Microbiol.* 12:631096. 10.3389/fmicb.2021.631096 33717021 PMC7953137

