# Supplementary figures and images for: Response of fermentation quality and microbial community of oat silage to homofermentative lactic acid bacteria inoculation
Source: Front Microbiol. 2023 Jan 20;13:1091394. doi: 10.3389/fmicb.2022.1091394 (PMC9895785; doi:10.3389/fmicb.2022.1091394)

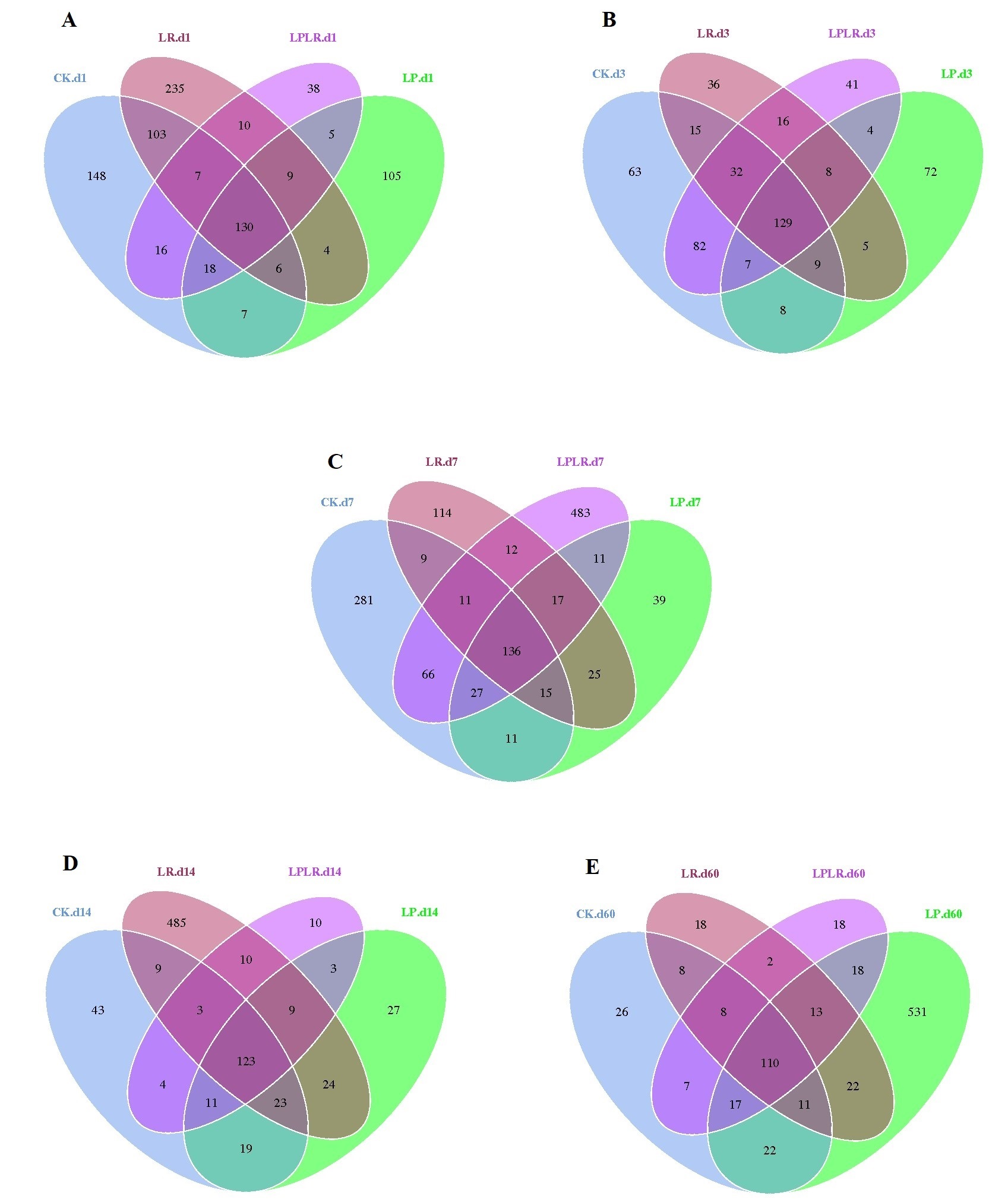

Supplement: Supplementary Figure 1 — Venn diagram depicting the common or unique bacterial OTU of oat silages at different days of fermentation: (A) 1 day; (B) 3 days; (C) 7 days; (D) 14 days; and (E) 60 days. CK, sterilized water; LP, Lactobacillus plantarum 694; LR, Lactobacillus rhamnosus 753; LPLR, 50% LP + 50% LR; d, ensiling days. [file Image_1.JPEG]

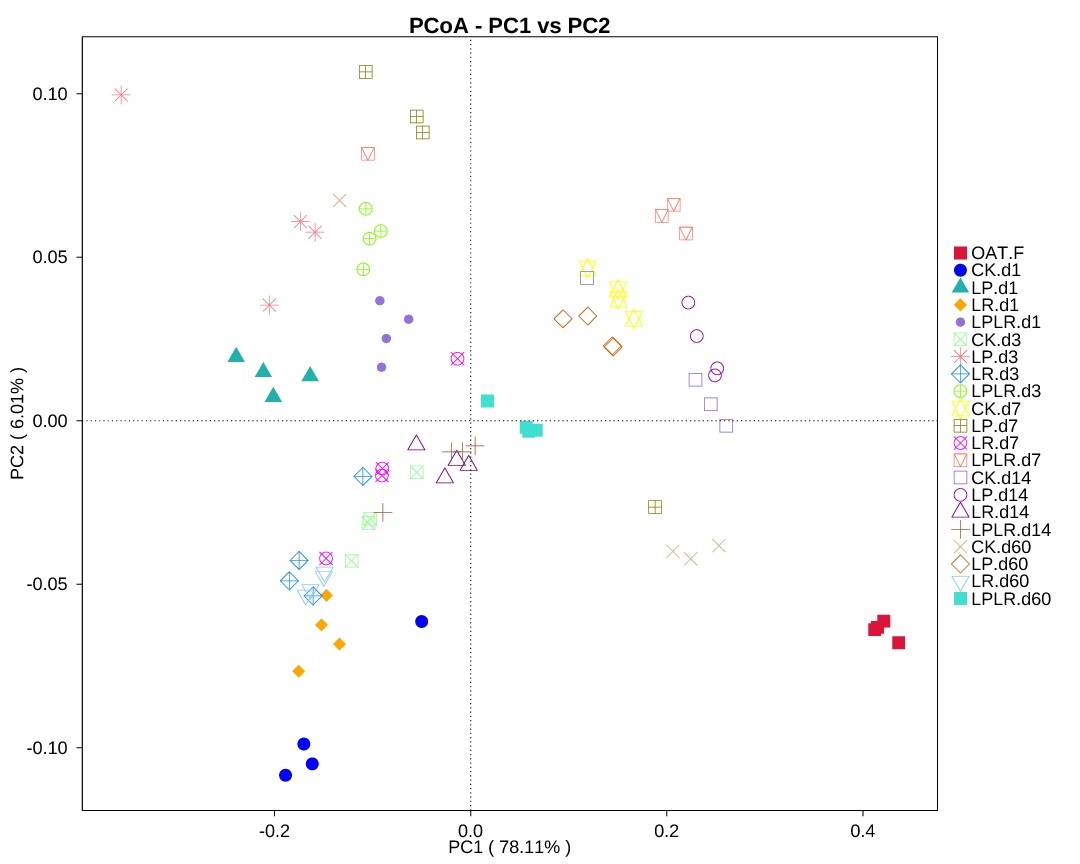

Supplement: Supplementary Figure 2 — Principal component analysis of bacterial community during ensiling at different days of fermentation with and without lactic acid bacterial inoculation. CK, sterilized water; LP, Lactobacillus plantarum 694; LR, Lactobacillus rhamnosus 753; LPLR, 50% LP + 50% LR; d, ensiling days; OAT.F, fresh oat. [file Image_2.JPEG]
